# Supplementary material for: Enhancing HIV-1 Neutralization by Increasing the Local Concentration of Membrane-Proximal External Region-Directed Broadly Neutralizing Antibodies
Source: J Virol. 2022 Dec 21;97(1):e01647-22. doi: 10.1128/jvi.01647-22 (PMC9888200; doi:10.1128/jvi.01647-22)
Supplement: Supplemental file 1 — Fig. S1 and S2 and sequences of 10E8 variants and D5_AR. Download jvi.01647-22-s0001.pdf, PDF file, 0.2 MB [file jvi.01647-22-s0001.pdf]

Supplementary information

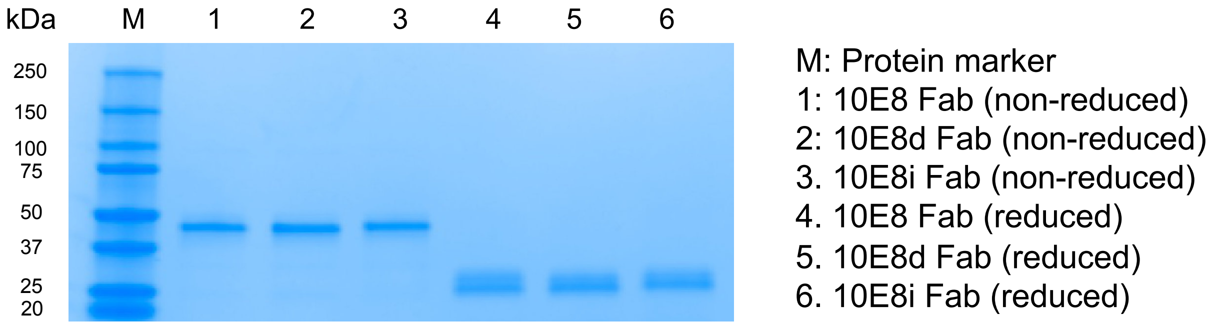

Fig. S1. Generation of 10E8 and its variants as Fab fragments. 10E8 and its variants (1 μg) were subjected to SDS-polyacrylamide gel electrophoresis and staining to visualize protein bands. Lane 1, non-reduced 10E8 Fab; lane 2, non-reduced 10E8d Fab; lane 3, non-reduced 10E8i Fab; lane 4, reduced 10E8 Fab; lane 5, reduced 10E8d Fab; lane 6, reduced 10E8i Fab. M, molecular weight marker.

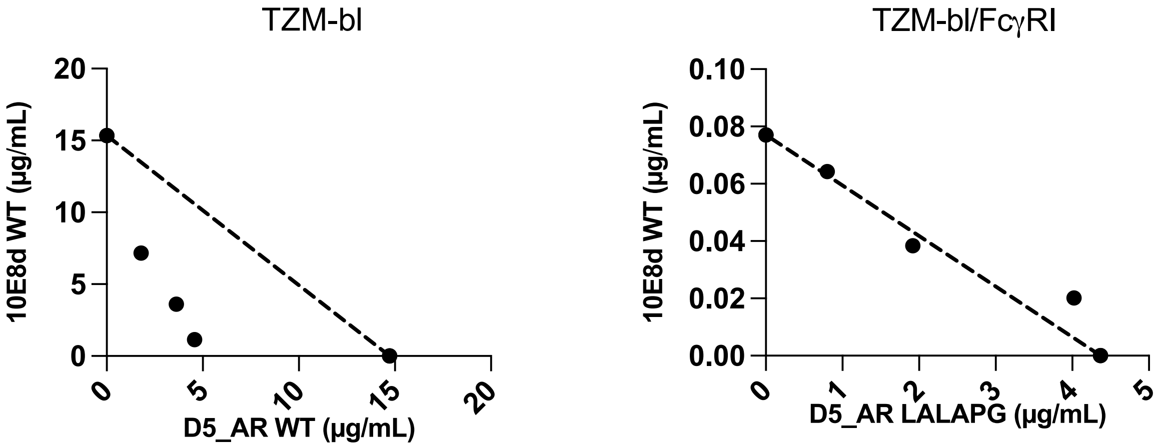

Fig. S2. FcγRI-bound 10E8d abolishes synergy with D5_AR. Isobologram analyses on the combination of 10E8d and D5_AR against viruses pseudotyped with Env from HIV-1 strains HXB2 in TZM-bl cells (left) and TZM-bl/Fc*γ*RI cells (right). The dotted lines indicate lines of additivity. Results are shown as mean ID_{50} performed in duplicates. Similar results were obtained in an independent repeat experiment. Data points below, along and above the line of additivity indicate synergy, additivity and antagonism, respectively.

Sequences:

10E8 light chain

SYELTQETGVSVALGRTVTITCRGDSLRSHYASWYQKKPGQAPILLFYGKNNRPSGV
PDRFSGSASGNRASLTISGAQAEDDAEYYCSSRDKSGSRLSVFGGGTKLTVL

10E8i light chain (mutations differing from 10E8 light chain underlined)

SYELTQETGVSVALGRTVTITCRGDSLR**R**HYASWYQKKPGQAPILLFYGK**R**NRPSGV
PDRFSGSA**R**GNRASLTISGAQAEDDAEYYCSSRDKSGSRLSVFGGGTKLTVL

10E8d light chain (mutations differing from 10E8 light chain underlined)

SYELTQETGVSVALGRTVTITCRGDSL**A**SH**A**ASWYQKKPGQAPILLFYGKNNRPSGV
PDRFSGSASGNRASLTISGAQAEDDAEYYCSSRDKSGSRLSVFGGGTKLTVL

10E8v4 light chain

SELTQDPAVSVALKQTVTITCRGDSLRSHYASWYQKKPGQAPVLLFYGKNNRPSGIP
DRFSGSASGNRASLTITGAQAEDEADYYCSSRDKSGSRLSVFGGGTKLTVL

D5_AR light chain

DIQMTQSPSTLSASIGDRVTITCRASEGIYHWLAWYQQKPGKAPKLLIYKASSLASGA
PSRFSGSGSGTDFTLTISSLQPDDFATYYCQQYSNYPLTFGGGTKLEIK

10E8 heavy chain

EVQLVESGGGLVKPGGSLRLSCSASGFDFDNAWMTWVRQPPGKGLEWVGRITGPG
EGWSVDYAAPVEGRFTISRLNSINFLYLEMNNLRMEDSGLYFCARTGKYYDFWSGY
PPGEEYFQDWGRGTLVTVSS

10E8v4 heavy chain

EVRLVESGGGLVKPGGSLRLSCSASGFDFDNAWMTWVRQPPGKGLEWVGRITGPGE
GWSVDYAESVKGRFTISRDNTKNTLYLEMNNVRTEDTGYYFCARTGKYYDFWSGY
PPGEEYFQDWGQGTLVIVSS

D5_AR heavy chain

QVQLVQSGAEVRKPGASVKVSCKASGDTFSSYAISWVRQAPGQGLEWMGSIIPLFGT
AAYAQKFQGRVTITADESTSTAYMELSSLRSEDTAIYYCARDNPTFGAADSWGKGT
LVTVSS
